# The role of lymphangiogenesis and angiogenesis in tumor metastasis

**DOI:** 10.1007/s13402-016-0281-9

**Published:** 2016-04-28

**Authors:** Roman Paduch

**Affiliations:** 1 Department of Virology and Immunology, Institute of Microbiology and Biotechnology, Maria Curie-Skłodowska University, Akademicka 19, 20-033 Lublin, Poland; 2 Department of General Ophthalmology, Medical University of Lublin, Chmielna 1, 20-079 Lublin, Poland

**Keywords:** Angiogenesis, Lymphangiogenesis, Lymph nodes, Metastasis

## Abstract

**Background:**

Metastasis is the main cause of mortality in cancer patients. Two major routes of cancer cell spread are currently being recognized: dissemination via blood vessels (hematogenous spread) and dissemination via the lymphatic system (lymphogenous spread). Here, our current knowledge on the role of both blood and lymphatic vessels in cancer cell metastasis is summarized. In addition, I will discuss why cancer cells select one or both of the two routes to disseminate and I will provide a short description of the passive and active models of intravasation. Finally, lymphatic vessel density (LVD), blood vessel density (BVD), interstitial fluid pressure (IFP) and tumor hypoxia, as well as regional lymph node metastasis and the recently discovered primo vascular system (PVS) will be highlighted as important factors influencing tumor cell motility and spread and, ultimately, clinical outcome.

**Conclusions:**

Lymphangiogenesis and angiogenesis are important phenomena involved in the spread of cancer cells and they are associated with a poor prognosis. It is anticipated that new discoveries and advancing knowledge on these phenomena will allow an improvement in the treatment of cancer patients.

## Introduction

It has firmly been established now that high mortality rates in cancer patients are not only associated with the occurrence of primary tumors but, even more profoundly, with the occurrence of metastases [1–3]. This notion implies that cancer-related death may not just be caused by distortion of the primary affected organs, but also by the distortion of organs at secondary sites, which jointly affect the whole organism. To initiate metastasis, a solid tumor that develops at a primary site may spread using existing routes that are related to normal body functions. During progression from an in situ tumor, aggressive malignancies may disseminate either via blood vessels (hematogenous spread after neovascularization) or via the lymphatic system (lymphogenous spread after lymphangiogenesis) [4]. Other ways through which tumor cells may spread include local tissue invasion and direct seeding into body cavities. Which way tumor cells choose to spread depends on the site of tumor initiation, the aggressiveness of the tumor cells, extrinsic signals and intrinsic tumor micro-environmental conditions including direct cell-cell and cell-matrix interactions within a tumor niche and the presence of paracrine factors mediating the formation of new vessels [5]. It has been found that solid tumors frequently induce angiogenesis and lymphangiogenesis. Blood and lymphatic vessels, however, offer diametrically different conditions for the migration and survival of tumor cells. These conditions are closely related to the distinct functions and structural features of these two systems [6].

## The functions and structures of blood and lymphatic vessels

The main function of blood vessels is to transport oxygenated blood, to exchange oxygen, carbon dioxide, water and mineral salts between blood and tissues and to regulate the pressure of the flow in the closed system powered by the heart. The lymphatic system, on the other hand, begins in peripheral tissues with blind-ended capillaries and has an open, semicircular layout. Lymph flows unidirectionally from the peripheral tissues to the blood. It does not carry oxygen or essential nutrients. Its primary function is to absorb extravasated protein-rich fluids, lipids, macromolecules and immunocompetent cells from the interstitial spaces within tissues. After resorption from the initial ducts (lymphatic capillaries and then pre-collectors), lymph is transported to larger vessels (lymphatic collectors and trunks) and flows back into the bloodstream mainly via the left lymphatic duct (thoracic duct). Normal functioning lymphatic vessels thus maintain plasma volume, prevent increases in tissue pressure and allow easy passage of leukocytes, thereby playing an important role in the proper functioning of the immune system and the immune surveillance of the whole body [4, 7–9].

Although blood and lymphatic vessels share a common embryological origin, they differ significantly. A first essential difference is the anatomy of lymphatic and blood vessels. Initial and terminal lymphatic capillaries are 10–60 μm in diameter and are lined with a layer of lymphatic endothelial cells (LECs) [10]. Blood capillaries are approximately 5–20 μm in diameter and have a uniform, compact layer of endothelium. Lymphatic capillaries, unlike blood capillaries, have an incomplete discontinuous basal lamina, or no basal lamina at all, and lack pericytes and smooth muscle cells [4, 8, 10, 11]. An important feature of lymphatic capillaries is the size of their lumen, which is three-fold wider than that of blood capillaries. Moreover, lymphatic capillaries are unique in that they have reticular, elastic and collagen fibers (anchoring filaments), which bind LECs to the extracellular matrix (ECM), a property that is vital to a proper lymph flow. The fibers stretch to open the lymphatic lumen (intracellular space) when the volume of the interstitial fluid increases, thus producing hydrostatic pressure. After this, the interstitial fluid flows into the lymphatic system causing the capillaries to dilate rather than to collapse [4, 7, 10–12]. This is a major feature of lymph absorption. The lymphatic collectors and trunks differ, however, structurally form lymphatic capillaries and are histologically similar to veins. They have a thin, three-layered coat and valves at the jugulosubclavian junction, which prevents blood reflux to lymphatic ducts as also retrograde lymph flow [8, 10, 13]. Normal blood vessels also contain three layers. The innermost layer (*tunica intima*) consists of a single layer of endothelial cells surrounded by connective tissue called the internal elastic lamina. The middle layer (*tunica media*) is built up of a basement membrane and smooth muscle cells surrounded by the external elastic lamina. The outermost layer (*tunica adventitia*) consist of connective tissue containing nerves that innervate the vessel [14]. Pathological veins that are formed as a result of tumor neovascularization differ significantly from normal veins. They are characterized by a chaotic structure in which endothelial cells do not adhere tightly to each other but, instead, form protrusions towards the lumen of the vessel. Also the pericytes adhere only loosely to the endothelium. The basal lamina is thinner than in normal vessels and has many fenestrations, which makes the vessels permeable. The diameter of the vessels may vary and blood clots can be formed, which may result in local differences in blood pressure. Increases in blood pressure and a high permeability of the vessels may result in the appearance of exudates, which prohibit the intravasation of cells. A chaotic organization of the vessel networks with numerous blind-ended vessels may lead to blood stasis, or even backflow [15, 16].

## A passive or active model of tumor cell dissemination?

Taking the above considerations into account, it appears that the lymphatic vessel pathway provides a better and safer route for cancer cell dissemination than the blood vessel pathway. The composition of the lymph fluid is almost identical to that of the interstitial tissue fluids, which promotes the survival of migrating tumor cells. Moreover, the discontinuous structure of the lymph capillary elements, a low lymph flow, a minimalized shear stress and a high concentration of hyaluronic acid, which plays an important role in cell protection and survival, give the lymphatic system an advantage over the bloodstream, in which mechanical forces and the toxicity of pure serum may negatively affect circulating tumor cells [7, 17]. Nevertheless, before an unequivocal claim can be made as to which route of dissemination is more effective, two questions need to be answered. The first one deals with the energy that metastasizing cells require for migration, i.e., whether it is more energy-efficient for cells to actively move to secondary sites or be shed passively. The second question is whether it is more proficient, from the cell’s point of view, to choose the hematogenous or the lymphatic pathway for the effective formation of distant micro-metastases. Lymphatic vessels are relatively leaky compared to blood vessels and, as such, are considered essential for tumor cell spread. The structure of blood vessels would, under normal circumstances, force tumor cells to spend more energy during intra- and especially extravasation. In the tumor neovasculature, however, many abnormalities can be found that actually facilitate tumor cell migration, including disorganized wall structures, endothelial fenestrations and a thin or even non-existing basement membrane [18]. The fragile new blood vessels that vascularize primary tumors are, to a certain extent, comparable to leaky lymphatic capillaries that lack a continuous basement membrane and contain many open junctions and pores. This open blood vessel structure suggests that tumor intravasation may be a passive process. However, it is well documented that CD73, an enzyme converting AMP to adenosine, is actively involved in vascular permeabilization. And although the ATP metabolism differs in blood and lymphatic vessels, it has been found that CD73 also plays an important role in normal lymphocyte migration into lymph nodes [19]. This enzyme may be considered as a major factor in the metastasis of tumor cells. Initially, tumor cells grow in their primary niche, but when the tumor reaches a diameter of about 1 mm, it invades its microenvironment and collapses the newly formed tumor blood vessels. Their fragile non-linear/disordered structure gives way under the pressure of the tumor mass, enabling passive entry of tumor cells into the vessel’s lumen and, subsequently, metastasis [20]. On the other hand, it has been shown that invasive tumor cells may detach form their primary masses and enter tumor-associated absorbing lymphatic (TAAL) vessels through intra-endothelial channels (1.8–2.1 μm in diameter), thus taking a passive route into the lymphatic circulation [21]. To simplify this model, tumor cells may be washed out from the tumor mass and, with the tide of tissue fluid, be pushed into lymphatic drainage canals, thereby initiating invasion. The passive model of intravasation is supported by the finding that most shed cells are non-vital and non-clonogenic. This means that the only cells that can survive in vessels (i.e., withstand the mechanical stresses and the attack of immune cells) and form metastases are tumor stem cells and cells that express a metastatic phenotype. However, such an argument implies the acceptance of an active mechanism of metastasis [21–23]. Other arguments in favor of an involvement of active mechanisms in the initial steps of metastasis include the accumulation of mutations, changes in the expression of adhesion molecules, the presence of chemokine gradients, expression of the urokinase plasminogen activator (uPA) followed by activation of metalloproteinases (MMPs), and the supportive role of stromal cells and cancer-associated fibroblasts, CAFs [24]. It is believed that alterations in the primary tumor microenvironment/niche induce tumor cells to migrate towards blood or lymphatic vessels. Active migration and entry into the vessels may also be facilitated by changes in the organization of the cytoskeleton as well as the acquisition of a metastatic phenotype through epithelial-mesenchymal transition (EMT) [23].

Despite many reports in the literature on this subject, there is currently no unequivocal answer to the question which conditions determine the choice of an active or a passive route of migration by tumor cells. It seems that, depending on the type of tumor, the stage of its development and/or the metastatic target organ, tumor cells may select either one or both of these routes.

## The role of epithelial-mesenchymal transition in tumor cell metastasis

Besides the acquisition of specific genomic changes by tumor cells that enable their growth and survival, epithelial-mesenchymal transition (EMT) and the reverse mesenchymal-epithelial transition (MET) are generally considered as some of the most fundamental processes underlying cancer cell dissemination. EMT is usually reported as being essential for the initial stages of malignant transformation and tumor development at the primary site, whereas MET is believed to be pivotal for the later, metastatic stages and for the formation of secondary tumors at distant sites [25]. Tumor cells undergo “type III EMT”, which differs from “type II EMT” that occurs during inflammation or fibrosis and “type I EMT”, which takes place under normal physiological conditions such as wound healing [26–28]. During EMT tumor cells acquire a mesenchymal, migratory and invasive phenotype by losing their intercellular junctions (i.e., adherens junctions, tight junctions, desmosomal junctions and also, partially, gap junctions), typical molecular markers (E-cadherin, cytokeratins), cytoskeletal organization (changes in microtubules, actin filaments, β-filamin or talin), apical/basolateral polarity and, finally, contact inhibition [27, 29–32]. At their primary site, tumor cells receive EMT promoting signals from activated stroma, while at metastatic sites such signals are weak or absent. Under the latter circumstances, metastatic tumor cells convert to an epithelial phenotype via MET. This process is also believed to support tumor-normal cell interactions at distant, metastatic sites, and to be facilitated by the stimulatory activity of the target organ parenchyma that induces the re-expression of E-cadherin. As a consequence, connections can be formed between neoplastic and normal cells. Such connections are very important for tumor cell survival within the new microenvironment [32]. On the basis of direct and indirect, paracrine stimulations, tumor cells may enter a state of dormancy at the metastatic target site. Dormant cells are characterized by a low metabolic activity, a suppressed anoikis associated with the formation of cell heterotypic E-cadherin, and a resistance to cytostatics due to activation of the receptor tyrosine kinase ErbB4 and induction of the PI3K-Akt pathway [25, 33–35]. Obviously, this is a simplified model of EMT-MET, and there are other factors that significantly promote not only EMT-MET, but also lymphangiogenesis and angiogenesis. Among them, the most important ones are α-SMA, whose increased expression by myofibroblasts has been associated with a high expression of N-cadherin, a LYVE-1-positive vessel count, and increased expression of VEGF, stromal cell-derived factor 1 (SDF-1) also known as C-X-C motif chemokine 12 (CXCL12), insulin-like growth factor-2 (IGF-2), hypoxia-inducible factor-1α (HIF-1α), transforming growth factor β (TGF-β) and hepatocyte growth factor (HGF) [30, 36–39]. In addition, immune cells such as macrophages, myeloid-derived suppressor cells (MDSCs), mast cells and neutrophils may contribute to EMT/MET transitions and the formation of new vessels through the production of cytokines, growth factors or proteases [30]. On the other hand, there are also molecules that may inhibit tumor angiogenesis, lymphangiogenesis and invasion. One such molecule is KAI-1/CD82, which belongs to the tetraspanin family of proteins and is considered to be a metastasis suppressor. This molecule is localized on the cell membrane and interacts with integrins and chemokines responsible for the adhesion, signaling and mobility of cells. Decreased levels of KAI-1/CD82 have been linked to limited cancer cell invasiveness and the suppression of metastatic cell growth mainly through inhibition of β-catenin-mediated EMT [40, 41]. Generally, molecules that are involved in EMT/MET transitions can be classified as EMT inducers (upstream cytokines and growth factors or receptors that initiate transition), EMT regulators (downstream transcription factors controlling the transition process) and EMT effectors (molecules that cause cell phenotype changes and endow cells with an invasive character) [32, 42].

Although EMT and MET are widely recognized as being essential for tumor cell metastasis, they may proceed differently in different cancers due to diverse characteristics of the respective blood and lymphatic vessel systems. It has e.g. been shown that prostate and breast cancers isolated form sentinel lymph nodes exhibit an increased invasive potential without any upregulation of mesenchymal markers [43, 44]. This finding confirms the concept that although mesenchymal transition contributes to the successful metastasis of tumor cells, lymph node dissemination does not per se require EMT [45]. This notion may be explained by the fact that the structure of the lymphatic system does not force mesenchymal cells to increase their invasive phenotype. Moreover, EMT facilitates intravasation, whereas cells leaving the vessels (extravasation) do not require this process [46]. Therefore, it may be concluded that in contrast to blood vessel dissemination, successful lymphatic migration may not require EMT. As such, EMT does not always appropriately imply the invasion of tumor cells and, although crucial, it is only one of the possible mechanisms underlying tumor cell metastasis.

## The role of the extracellular matrix in tumor cell metastasis

The tumor stroma is composed of non-cellular components of the extracellular matrix (ECM) such as proteins, glycoproteins, proteoglycans and polysaccharides endowing complex physical and biological properties to the stroma, as well as immune cells, endothelial cells and fibroblasts (CAFs) that participate in the early stages of tumor cell dissemination [47, 48]. The ECM, besides its structural and biomechanical features, can also function as a repository for active components, such as growth factors, which are stored and released during alteration or remodeling of its composition. Some of the most important constituents of the ECM are laminins, which have a significant impact on cellular dynamics, and collagens, which are the major structural components of the matrix [49–51]. Reorganization of these and other matrix constituents during cancer development results in deregulation of the ECM and disruption of its integrity and architecture, thereby promoting epithelial cell transformation and tumor cell progression [48]. Deregulated ECM dynamics are closely related to the expression and activity of ECM enzymes including MMPs, heparanases, 6-O-sulfatases, cysteine cathepsins, urokinase and the serine protease plasmin [52, 53]. This deregulation leads to essential changes in ECM properties, which not only may induce tumor cell motility, but may also affect tumor micro-environmental stromal cells such as CAFs, immune cells and mesenchymal stem cells (MSCs) [54–57].

It has been found that released or plasma fibronectins play an important role in tumor cell adhesion, migration, invasion and survival by activating integrins via the MAPK/ERK pathway [58]. Other ECM components and receptors, including heparan sulfate proteoglycans and CD44, may facilitate the growth and motility of tumor cells [59–61]. An abnormal ECM has also been found to participate in the induction of angiogenesis and lymphangiogenesis during tumor progression. It has been reported that fragments of type IV and type XVIII ECM collagens, including endostatin, tumstatin, canstatin, arresten and hexastatin, strongly influence angiogenesis, either directly or indirectly by modulating the VEGF level [62]. They may also modify lumen entrapment involved in tube formation during angiogenesis. [63, 64]. The role of the ECM in lymph vessel formation is as yet poorly recognized, but it has been shown that the ECM receptor integrin α9β1 may be involved in the induction of tumor lymphangiogenesis [65, 66]. Similarly, low molecular weight hyaluronian (LMW-HA) has been found to promote lymphangiogenesis via interactions with its lymphatic vessel endothelial hyaluronian receptor 1 (LYVE-1), which leads to the induction of LEC proliferation and tube formation [67]. Recently, it has also been found that MT1-MMP-mediated proMMP-2 activation and the expression of ECM1 soluble protein and EMILIN1, an elastic microfibryl-associated protein, are closely linked to lymphangiogenesis and tumor invasion [68–70]. Taken together, it may be concluded that changes in biochemical and biomechanical properties of the ECM represent important factors affecting tumor cell behavior during metastasis.

## How do tumor cells choose between blood and lymphatic vessels for dissemination?

If tumor cells can use passive and active mechanisms of intravasation, the question arises what the basis is of dissemination via blood and lymphatic vessels. It has already been reported that carcinomas and melanomas are more likely to form lymph node metastases than sarcomas [46]. It is unclear, however, why, when or where the decision about the route of intravasation is made. Some hypotheses on this matter have already been put forward and all of them appear to be equally probable. The most obvious factors that may be involved in this process are the physical/mechanical conditions and the genetic or epigenetic programs that are innate to tumor cells. Also, the role of factors that attract tumor cells towards blood or lymphatic vessels should not be ignored. Among them are inflammatory and host hematopoietic precursors, but also soluble factors such as chemokines, growth factors and soluble receptors [71]. They not only affect the metastatic phenotype of tumor cells, but also in cases where due to various reasons (e.g. receptor mismatch, difficulties with EMT, a migration mode that makes it difficult to penetrate the blood wall barrier) tumor cells are unable to cross blood vessels, they direct them into the peri-tumoral lymphatic system (metachronous seeding), which provides a safer and easier way for the cells to intravasate and disseminate. As an example, it has been reported that bradykinin may act as a signal that attracts glioma cells to blood vessels [72]. Subsequently, these tumor cells may establish so-called satellite lymph node metastases, which disseminate metastatic cells via the thoracic duct. Some authors claim that lymph nodes select tumor cells, enabling those with a high enough malignant phenotype to disseminate further. They explain *ipso facto* the differences in malignancy between primary tumor cells and metastatic tumor cells [46]. Following this idea, it seems obvious that inhibition of lymph node metastasis should inhibit hematogenous spread. Experimental data show, however, that this is not always the case. Moreover, it has been reported that distant metastases can be formed despite a lack of metastatic cells in sentinel and distant lymph nodes. This, in turn, may confirm direct dispersal of tumor cells into blood vessels. There is also a model which proposes that tumor cells may stay for some time in a non-metastatic state. This state lasts until the cells are activated and recruited to disseminate simultaneously via blood and lymphatic vessels [46]. This hypothesis may explain the quick and massive metastasis which is characteristic for some cancers.

Tumor cells may disseminate via blood or lymphatic vessels, but do they show a “predilection” for one route of migration over the other? Such predilection may depend on various factors that are specific for the tumor cells, as well as for their microenvironment and the newly formed vessels. In addition, specific molecular signaling pathways may play a major role. Differences in gene expression between the lymphatic and blood endothelium may constitute one of the major factors that is decisive for the route of dissemination that tumor cells choose. Blood endothelial cells (BECs) typically express CD44, ICAM1, Tie-2/Ang-1 VEGFR-1 and -2, Neutropilin-1 receptors for VEGF-A, -C and -D, and secrete IL-6/8 and MCP-1. On the other hand, lymphatic endothelial cells express c-Met/HGF, Tie-2/Ang-1/2, IGF-Rs/IGF-1/2, FGF-Rs/FGF-2, Podoplanin, LYVE-1 and VEGFR-2 and -3, receptors for VEGF-C and -D [73–75]. The role of these factors is widely accepted now, despite controversies on the role of VEGF-D in lymphangiogenesis and tumor cell dissemination via lymphatic vessels in some cancers, such as ovarian and breast cancers [76–79]. VEGF-D has been reported to act as a factor that induces both intra- and peri-tumoral lymphatic vessel development, but not necessarily lymph node metastasis [80, 81].

**Fig. 1:**
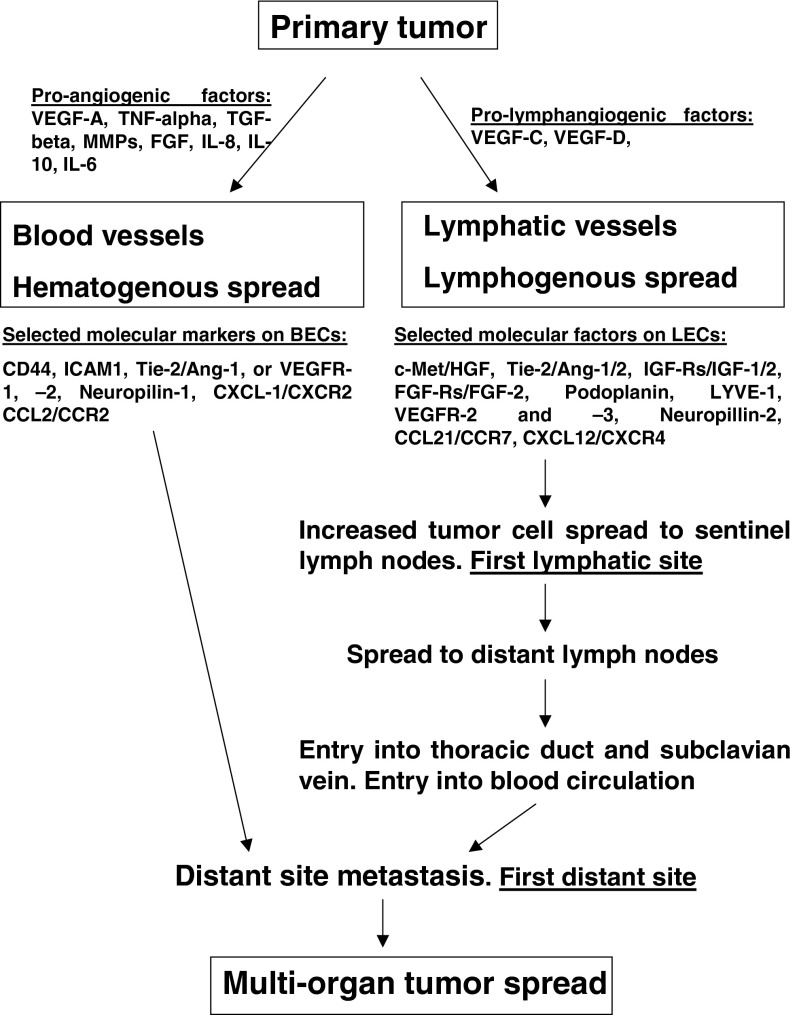
Routes of cancer cell spread. Metastatic cells may enter directly into blood vessels (hematogenous spread) that vascularize the tumor mass and, in this way, disseminate to distant sites. Another trail of cancer cell spread may be the penetration into lymphatic vessels (lymphogenous spread) and dissemination via the lymph flow to sentinel and, subsequently, distant lymph nodes. Next, the cells may enter the thoracic duct, the subclavian vein and, ultimately, distant sites

Gene expression profiles may not only differentiate the properties of the two cell types involved (i.e., BECs and LECs), but also the physiological functions of blood and lymphatic vessels and their potential to be selected by tumor cells as a route for metastasis [74]. On the other hand, selection pressure can also be exerted on tumor cells through the expression of different receptors and signaling molecules by the lymphatic or blood endothelium, which allows cells to transmigrate via the blood or lymphatic vessel linings only, depending on what specific co-receptors the tumor cells express. It has also been suggested that the choice between lymphangiogenesis and angiogenesis may depend on the ratio of the different inducing factors present within the local tumor microenvironment [82]. Also, crosstalk between lymphatic and blood endothelial cells, as well as between endothelial cells and the vessel milieu, should not be ignored as important aspects in the selection of one of the two routes of tumor cell dissemination [80]. It appears that the ultimate selection depends on several factors, including the specific structure and mechanical functionality of the vessels as also the expression of adhesion molecules, the secretion of chemokines and the activity of specific signaling pathways. Which pathway is chosen depends on the concentration of local factors at the primary site as also at the site of the metastatic niche, the tumor cell of origin, the stage of tumor development and, conceivably, the patient’s health status. It seems most probable that both routes may be involved in metastasis, but not necessarily at the same time (Fig. 1).

## Are lymphatic vessels developed during metastasis?

For a long time scientists were convinced that only blood vessels, which drain the whole body, are able to transport cancer cells to secondary, metastatic sites. This view changed when direct and indirect paracrine tumor-stroma interactions in the primary tumor niche, as well as intra-nodal lymphangiogenesis, were discovered [83]. It was found that tumor cells can alter the surrounding microenvironment and, thus, influence tumorigenesis through crosstalk with dendritic cells, CAFs, macrophages, lymphocytes and pericytes, all of which can secrete soluble molecules that exhibit angiogenic or lymphangiogenic activities [84]. These molecules may, in turn, stimulate the enlargement of tumor lymphatic vessels, thereby facilitating cancer cell invasion. Another milestone was the introduction of the lymphvascular niche concept [85]. This concept may explain the role of lymphatic vessel formation within nodes, thereby constituting an intermediate platform for the lymphatic metastasis of cancer cells. Indeed, it has experimentally been shown that primary tumors can induce lymphatic vessel formation (neo-lymphangiogenesis) within the tumor draining lymph node [86]. This process precedes metastasis, possibly indicating that primary tumors may first generate a favorable microenvironment at this site for a subsequent preferential and successful dissemination. This lymphvascular niche may not only attract spreading tumor cells but also a subset of cancer stem cells (CSCs), which are the potential initiators of secondary tumor development at distant sites [87–89]. This notion is also of relevance for clinicians who use resected primary tumor samples and regional lymph nodes to determine the stage of the disease, the most optimal treatment regimen and the patient’s prognosis.

## What is the role of lymphatic and blood endothelial cells in metastasis?

**Fig. 2:**
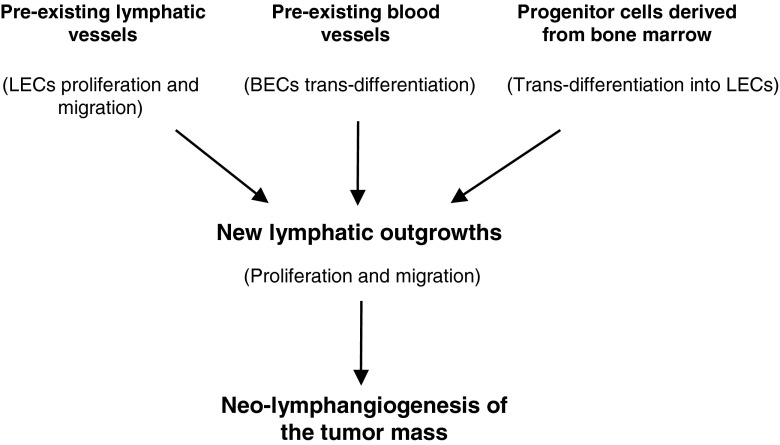
Origin of lymphatic endothelial cells. Endothelial cells that form neo-lymphatic vessels may originate from three alternative sources. First, they may originate from pre-existing lymphatic vessels in which lymphatic endothelial cells (LECs) proliferate and migrate, resulting in outgrowths that vascularize the tumor mass. Second, they may originate from pre-existing blood vessels in which blood endothelial cells (BECs), through the action of lymphatic growth factors, trans-differentiate into lymphatic endothelial cells. Third, they may originate from progenitor bone marrow-derived cells that, after recruitment to sites of lymphangiogenesis, in the presence of specific growth factors undergo trans-differentiation into lymphatic endothelial cells

Another important point is the origin of the endothelial cells that form tumor lymphatic vessels. As yet, three potential sources of lymphatic endothelial cells (LECs, Fig. 2) have been reported [87]. The first one is a pre-existing lymphatic vessel in which, after appropriate stimulation, LECs proliferate and migrate leading to neo-lymphatic outgrowth. Subsequently, the tumor cells and the tumor stroma induce the formation of new lymphatic capillaries by secreting VEGF-C and other cytokines and chemokines. Subsequent interactions with specific receptors induce LEC-based tube formation by stimulating cell proliferation and longitudinal growth. Thus, tumor LECs may be induced locally, i.e., be derived from local vessels in specialized parts of the lymphatic system [87, 90]. A second route for the formation of tumor LECs is the transdifferentiation of endothelial cells from pre-existing blood vessels. In this process VEGF-C plays an essential role, as also the lymphatic-specific receptor VEGFR-3, which is expressed in blood vessels in tumors, and the key transcription factors SOX18, COUP-TFII and PROX-1 [87, 90, 91]. Likewise, it has been shown that integration of circulating cells that exhibit lymphendothelial features may initiate a pathologic outgrowth of the lymphatic system. Thus, a third source of tumor LECs comes from transdifferentiation of non-endothelial cells [92]. Although bone marrow-derived cells (BMDCs), including endothelial progenitor cells, are essential for the formation of new blood vessels, it is conceivable that they may also contribute to neo-lymphangiogenesis in tumors. Their participation may not only be direct via transdifferentiation, but they may also play inducing or supporting roles under pathological conditions, without necessarily taking part in the lymphangiogenic process itself [93]. Other cells, such as mesenchymal stem cells (MSCs) or tumor associated macrophages (TAMs), may also participate in this process. Under specific conditions prevailing in tumor masses such as hypoxia, MSCs may differentiate into ECs, thereby contributing not only to angiogenesis but also to lymphangiogenesis [94]. TAMs, which belong to the myeloid lineage, are multifunctional cells that, depending on the micro-environmental conditions, may switch their phenotype and functionality. They can transdifferentiate and structurally act as ECs, i.e., form cellular elements of the lymphatic vessel wall [95]. On the other hand, macrophages also constitute a rich source of bioactive molecules that, under appropriate conditions such as tumor inflammation, can be released by the activated cells. Among these molecules are VEGF-A/-C/-D, which initiate lymphangiogenesis both through the stimulation of LEC proliferation and the consecutive recruitment of TAMs. In this latter scenario, it is highly probable that both subtypes of macrophages (i.e., M1 and M2) may interact with tumor LECs and participate in pathologic lymphangiogenesis, either directly or indirectly in a paracrine fashion [87, 96–100].

In blood vessels, a similar role is played by blood endothelial cells (BECs). Both BECs and LECs are endothelial cells, so it may be reasonable to assume that these two types of cells do not differ significantly. In conformity with the diverse roles played by these cells in blood and lymphatic vessels, however, they exhibit clear differences in cell-cell and cell-matrix interactions. In fact, differences have been found not only in their arrangements in the respective vessels, but also in their responses to the micro-environmental signals that they receive and secrete (i.e., angiocrine and lymphangiocrine factors). These functional differences may obviously result from the interstitial flow conditions to which the ECs are exposed, but it has also recently been shown that an important role is played by the genes they express [101–104]. This is a third level at which the morphological features and the micro-environmental interactions of ECs may be programmed and controlled. Despite these differences, both BECs and LECs play important roles in blood and lymph flow and, under pathologic conditions, they may serve as enhancers of tumor cell adhesion to the vessel wall, the transmigration of tumor cells and, consequently, tumor progression.

## Do intra- and peri-tumor lymphatic vessels participate in tumor cell dissemination?

Another intriguing question is to what extent intra-tumor lymphatic vessels (ITLs) and peri-tumor lymphatic vessels (PTLs) participate in tumor cell dissemination. Two types of tumor lymphatic vessels can be distinguished on basis of their localization in the cancerous mass. Lymphatic vessels, both pre-existing and newly formed, can be found in the tumor periphery and inside the tumor mass and are, accordingly, called peri- and intra-tumoral lymphatic vessels, respectively [105]. Although their roles in tumor dissemination are different, they are both considered to be responsible for the formation of metastases. ITLs have been associated with a poor survival, whereas PTLs have been associated with the occurrence of nodal metastases and overall clinical outcome [106–108]. In primary colorectal tumors, immunohistochemical staining has revealed the presence of an extensive PTL network, which may be related to a relatively short time from tumor development to the formation of metastases [107]. PTLs are generally believed to uptake tumor cells and to facilitate their dissemination. ITLs are, on the other hand, usually small, compressed, collapsed and non-functional, due to increased mechanical forces related to tumor growth, the invasion of migrating tumor cells and an increased interstitial fluid pressure inside the tumor mass [87]. Therefore, ITLs may be considered as components within tumors that initiate and promote metastasis, but do not act as routes of direct cancer cell spread or enhanced lymph node metastasis (LNM). This role is attributed to PTLs, which surround the tumor periphery with functional vessels. By increasing the absorptive area, which collects fluid and tumor cells from the external layers of the cancer mass that mainly contain fast proliferating tumor cells, they promote lymphatic metastasis [109]. Such lymphatic vessels are sufficient for a cancer to spread, which is supported by the fact that tumors that lack ITLs are often still able to disseminate. Therefore, an increased PTL vessel density, especially in VEGF-C over-expressing tumors, is usually considered as a predictor of a high risk of lymphatic metastasis [110]. Additionally, when tumor cells penetrate PTLs they may, through direct interaction, stimulate the proliferation of normal LECs (NLECs) and, in this way, promote or enhance lymphangiogenesis. Obviously, paracrine influences of NLEC-derived lymphangiogenic factors are also important. Together, these factors may be potent enough to re-program NLECs to tumor LECs (TLECs), which exhibit different morphological, functional and molecular characteristics [1].

## Additional factors influencing tumor cell dissemination

Many additional factors are known to be strongly related to tumor cell dissemination and, consequently, a poor prognosis, including lymphatic vessel density (LVD), blood vessel density (BVD), interstitial fluid pressure (IFP) and tumor hypoxia. Although these factors are not unique to tumor dissemination and/or a poor prognosis, they may occasionally constitute a therapeutic problem.

### Lymphatic vessel density and blood vessel density

Lymphatic vessel density (LVD) is defined by the number of ITLs and PTLs per area. A high LVD may facilitate direct interactions between tumor cells and lymphatic vessels, thereby increasing the probability of invasion. In many tumors, a correlation has been found between a high LVD and the occurrence of lymph node metastases. Moreover, it has been found that in colonic carcinomas the number of tumor-associated lymphatic vessels may be increased compared to that in the normal tissue microenvironment [7, 111]. Such features predict an unfavorable prognosis [108]. However, it is still unclear whether a high LVD is a condition *sine qua non* for metastasis, or whether it only initiates and facilitates the spread of tumor cells. Another point that requires clarification is whether quantification of lymphangiogenesis can be used as a diagnostic criterion for early and late tumor stages. In addition, it should be established whether imaging of lymphatic vessels is sensitive enough to be used as a diagnostic and prognostic tool in cancer patients. Some studies support the usefulness of this approach, claiming that a high lymphatic micro-vessel density, but not the invasion of tumor cells into lymphatic vessels, may be considered as a biomarker that correlates with a poor clinical outcome [112–115]. The outcome of these approaches may, however, depend on the conditions and the assumptions made for the clinical tests. Therefore, in occasional tumors a low lymphatic micro-vessel density may be related to a high invasive capacity of the tumor cells and, as a consequence, an early dissemination. Clearly, further research is needed to validate either one of these hypotheses.

Blood vessel density (BVD) provides a measure of blood vessel development and remodeling in both the tumor microenvironment and the tumor mass itself [116]. BVD results from the activity of pro- and anti-angiogenic factors. It has been shown that besides VEGF, also other factors may affect blood micro-vessel expansion. The placental growth factor (PLGF) has for example been reported to affect cervical cancer BVD and, concomitantly, its progression and metastasis. Alternatively, PLGF mRNA expression has been found to also correlate with LVD [117]. Above (section 3) we noted that abnormal tumor blood vessel structures may be indicative of an ongoing metastatic process. The impact of BVD on the rate of tumor cell dissemination could, however, also be considered as a predictor of metastasis, similar to LVD. It has already been shown that LVD in conjunction with BVD may serve as an independent prognostic factor in colorectal carcinoma [118]. In general, it is likely that higher BVDs and higher LVDs will correlate with each other and with a higher probability of the formation of distant metastases. However, all these features may be related to the cancer type, as well as to the way the vessels are formed and the functional and structural characteristics of the newly formed vessels. With the current state of knowledge, the exact mechanisms underlying anomalous tumor vascularization, i.e., lymphangiogenesis or angiogenesis, remain to be resolved.

### Interstitial fluid pressure

It is well known that an abnormal blood or lymphatic vasculature, or in some cases the lack of a lymphatic system, may lead to an increase in interstitial fluid pressure (IFP) [119, 120]. Impaired lymph drainage and increased lymphatic permeability are the main factors that result in IFP alterations. Maximum IFP values have been observed in cases with a high micro-vascular density (MVD) due to a concomitant heterogeneity of the tumor vasculature and an uneven distribution of the vessels within solid tumors [119]. An elevated IFP is a barrier to tumor therapy, since it impairs the access of anti-tumor agents to the tumor mass and facilitates the entrance of transformed cells into the peripheral lymphatic vessels. This implies that tumors may be treated by reducing the MVD and, thereby, lowering the IFP [119, 121]. An elevated IFP may increase the number of dying cells within the tumor mass and induce the formation of abnormal blood and lymph vessels, due to over-expression of VEGF. This may, ultimately, result in increased tumor cell motility [96]. Based on this, IFP has been proposed as a potential biomarker for metastatic spread [122]. There are, however, also data indicating that high primary tumor IFP values may not correlate with a high metastatic rate. It has also been suggested that tumor IFP values may serve as biomarkers for treatment responses, rather than being the cause of metastasis [121].

### Tumor hypoxia

Another important causative factor and indicator of tumor dissemination is low oxygen tension (hypoxia). The effects of hypoxia are controlled by the activity of the transcription factor HIF-1 [123, 124]. HIF-1 is a heterodimer consisting of two subunits: a constitutively expressed β subunit and an oxygen-regulated α subunit. In colorectal cancer a significant correlation between VEGF-C and HIF-1α expression has been observed. The clinic-pathological consequences of this relation include lymphatic capillary formation and lymphatic liver metastasis [125, 126]. HIF-1 regulates the transcription of more than 70 genes, including those enhancing cellular metabolism and initiating angiogenesis and metastasis. HIF-1 inhibition prevents tumor initiation, progression and spread to distant organs via lymph and blood vessels, and limits resistance to therapy [127]. A special role in VEGF-C/HIF-1α interdependency is played by inflammation and by the accumulation and activity of TAMs in hypoxic areas. In response to a decreased oxygen tension and an inflammatory microenvironment, HIF-1α expression may become up-regulated in macrophages. This up-regulation enhances the expression of VEGF-A/-C which, in turn, induces LEC differentiation and proliferation. As a result, new lymphatic vessels are formed and pre-existing lymphatic vessels are re-modeled, thereby creating an opportunity for tumor cells to invade [125, 128, 129]. Similarly, HIF-1 has been found to act as a strong pro-angiogenic factor. It stimulates BEC proliferation, which contributes to the formation of new blood vessels [116]. On the other hand, it has been shown that decreased HIF-1α expression, due to up-regulation of a basic helix-loop-helix (bHLH) transcriptional repressor (SHARP1, bHLHE41 or DEC2), may inhibit tumor growth and angiogenesis via a negative regulation of VEGF expression [130].

Continuous re-modeling of blood vessels is the main reason for an unstable blood flow, which may induce cyclic hypoxias. HIF-1, which is induced by a low oxygen tension (below 1 %), stimulates glycolysis, angiogenesis, drug resistance, autophagy, proliferation of tumor cells and immunosuppression, as well as tumor cell motility. It appears that increased LVD, BVD and IFP in the primary tumor niche are all closely related to the induction of tumor hypoxia and should, therefore, all be considered as components of a metastasis-supporting microenvironment.

## Regional lymph node metastasis

Dissemination of cancer cells to regional lymph nodes is the first step in metastasis and, as such, serves as a useful tool for cancer staging and prognosis [131, 132]. In general, lymph node metastasis correlates with a poor prognosis. Other, auxiliary factors that predict a poor outcome include micro-lymphatic vessel density (MLD) and high expression levels of VEGF-C, CXCR4, Flt-4, VEGFR-3 and VEGF-D, which have been proven to contribute to lymphatic involvement and nodal metastasis [133–136]. Factors that regulate or alter the rate of lymphatic metastasis can be classified as endocrine, cytotoxic, anti-angiogenic, anti-inflammatory and immune modulatory. At a higher level, these factors can be divided into exogenous and endogenous, influencing stimulators or inhibitors of lymph node dissemination [103]. Whether lymph node metastases indeed fully correlate with a poor prognosis and whether they may constitute a prognostic value in predicting distant dissemination of tumor cells to other organs is still a matter of debate. On the one hand, it has been stated that lymph node metastasis may not be related to tumor aggressiveness and tumor cell migration to distant organs. This claim is based on two observations. Firstly, it has been found that lymphadenectomy may not provide survival benefits and that tumor cells from primary masses may exhibit a similar disseminating potential as those from lymph nodes. Secondly, it has been found that tumor cells that have entered and adapted to the lymphatic system may not be able to efficiently form organ-specific metastases [137]. An opposing view is that disseminating tumor cells may efficiently use lymph node blood vessels or efferent lymphatic vessels to spread to other parts of the body. In addition, dissemination to lymph nodes may also occur in the absence of typical lymphangiogenesis, since cancer cells may also employ pre-existing lymphatic vessels. Therefore, while the presence or absence of cancer-related lymphangiogenesis may depend on tumor type, migration into lymph nodes seems to be an indispensable element of effective metastasis [138]. The latter theories appear to closely reflect the actual situation, since a lack of nodes often means that no distant metastases can be formed, which indicates that transition of tumor cells via nodes is a prerequisite for further dissemination. Lymphangiogenesis and migration of tumor cells into lymph nodes seems to be a preferential, active process that is necessary for further dissemination. Tumor cell migration may also induce lymph node lymphangiogenesis (LNL). The concept of LNL suggests that lymphatic and distant metastases are closely connected with each other as well as with tumor-inherent behavior and local responses of the host immune system to tumor-derived stimuli [139].

## The primo vascular system as a possible conduit for metastatic cells

The primo vascular system (PVS) is a recently discovered novel circulatory system that may exist next to the lymphatic and blood vascular systems. Initially, vasculogenic mimicry was considered to represent an additional relevant vascular structure, but its primitive micro-circulation failed to provide an explanation for potential routes of tumor cell metastasis. Therefore, additional studies were performed that finally led to the discovery of a third vascular compartment currently known as the primo vascular system [140–142]. This system was found to be present throughout the whole body on organ surfaces, inside lymphatic and blood vessels and their sub-vessels, as well as on the surface, around and within subcutaneous tumors [140, 141, 143, 144]. This structure is anatomically composed of small primo vessels (PV) with diameters of 20–50 μm and primo nodes (PN) approximately 100–1000 μm in size [141, 143]. Because of its wide distribution, its high density in tumor masses and its connection with the tumor microenvironment, the PVS is currently considered as a potent route for cancer cell metastasis. Its role may be important especially since the PVS directly connects primary and secondary tumors and since cancer cells can actively be transported via this system [143]. Moreover, because the PVS has its own circulating fluid, which contains cells expressing stem cell markers (i.e., CD133, Oct4 or Nanog), it may play a role in the regeneration of cancer stem cells or serve as a unique niche for these cells [140, 143]. It may, therefore, be hypothesized that next to lymphatic and blood vessels, cancer cells use the PVS for effective dissemination and the formation of secondary tumors at distant sites. This hypothesis may at least partially explain the failure or ineffectiveness of previous and current clinical trials designed to inhibit metastasis by only suppressing lymphangiogenesis or angiogenesis [144]. It would also disprove the dogma of the existence of only two routes for cancer cell dissemination.

## Conclusions and future perspectives

Both in vivo animal models and in vitro wound healing assays have indicated that lymphangiogenesis occurs after angiogenesis [91, 145]. The formation of lymphatic vessels may, therefore, rely not only on lymphangiogenic but also angiogenic factors. The induction of lymphangiogenesis may also be affected by micro-environmental conditions at the primary tumor site, including typical physical or mechanical stresses. When angiogenesis occurs, the number of proliferating tumor cells within the solid tumor mass should be large enough to be able to mechanically expand the primary niche. In addition to that, lymphatic vessel formation may be induced/regulated by the hydrostatic pressure related to the newly formed and still leaky vessels. Although tumor lymphangiogenesis is considered to be secondary to angiogenesis, it may also occur independent from the formation of new blood vessels. Currently, it is believed that both systems play an equally vital role in tumor spread. This notion is substantiated by the fact that lymphatic and blood vessels are physically connected, and that tumor cells can enter the blood stream directly via venous capillaries or indirectly via lymphatic vessels.

Knowledge gained on the mechanisms of cancer metastasis should be transferred to clinical practice. One area of clinical application is based on the awareness that tumor spread depends on the production and availability of specific factors. It is now common knowledge that malignant carcinomas spread to distant organs via routes that the organism uses to supply its tissues with oxygen and nutrients. To survive and migrate to distant sites, cells within the tumor mass have to develop mechanisms that enable them to create connections with normal arteries in the surrounding tissues. The initiation of blood and lymphatic vessel formation in tumors is associated with the activation of specific paracrine factors present within the microenvironment. These factors are produced both by the tumor cells themselves and by their surrounding stroma. This knowledge is currently employed in therapeutic models that involve the specific blocking of selected molecules (i.e., cytokines, chemokines and growth factors) produced in abnormal quantities in cases with, or at the risk of developing, metastases. Also, targeted immunotherapy studies are currently aimed at blocking the formation of pathways of tumor cell spread. Another aspect of metastasis both via blood and lymphatic vessels that is important from a clinical point of view is related to the fact that disseminating tumor cells and tumor cells at distant sites may acquire resistance to chemotherapy, radiotherapy and apoptosis-inducing therapy. Knowledge on the mechanisms underlying the acquisition of these resistances can be used for the design of new clinical management strategies aimed at overcoming or preventing the development of these resistances.

In the future, the knowledge gained on the mechanisms underlying lymphangiogenesis and angiogenesis should not only allow a more effective treatment of cancer patients through e.g. the inhibition of metastasis, but should also give clinicians more effective tools for the prevention and prognosis of cancers based on the degree of tumor-associated vasculature development. The future clinical management of patients based on knowledge of tumor spread mechanisms may more profoundly rely on subtle interventions within the molecular pathways regulating lymphangiogenesis and angiogenesis, as well as the molecular pathways regulating tumor cell motility. As there is currently no explicit evidence available that one system is more efficient in cancer cell dissemination than the other, further research is needed to determine the exact role of both blood and lymph vessels in the development and metastasis of tumors of different origin.
